# Safety and efficacy of perioperative continuous renal replacement therapy for percutaneous coronary intervention in severe acute myocardial infarction patients

**DOI:** 10.25122/jml-2022-0270

**Published:** 2023-05

**Authors:** Hanxiang Gao, Ming Bai, Aiai Chu, Chenliang Pan, Jing Zhao, Zheng Zhang, Xiaofeng Shang

**Affiliations:** 1. Department of Cardiology, First Hospital of Lanzhou University, Lanzhou, China; 2. Department of Cardiology, Gansu Provincial Hospital, Lanzhou, China; 3. Department of Cardiology, Zhangye People's Hospital Affiliated Hexi College, Zhangye, Gansu, China

**Keywords:** continuous renal replacement therapy, acute myocardial infarction, percutaneous coronary intervention

## Abstract

This retrospective study aimed to evaluate the safety and efficacy of continuous renal replacement therapy (CRRT) during percutaneous coronary intervention (PCI) in patients with severe acute myocardial infarction (AMI). The study analyzed data from 945 AMI patients hospitalized between January 2016 and December 2017, out of which 21 patients underwent perioperative CRRT for PCI. We assessed the baseline characteristics of severe AMI patients before and after CRRT and examined the effect of CRRT on cardiac, renal, and liver function, as well as other indicators. The heart rate of patients undergoing CRRT was significantly lower at 24 h and 48 h after CRRT than before CRRT (p=0.038). There was a moderate but not significant decrease in the mean systolic blood pressure or diastolic blood pressure (p>0.05). Importantly, we found that significantly more patients showed Killip class I-II and significantly improved cardiac function after CRRT (23.8% vs. 57.1%, p=0.001). The levels of urea nitrogen, creatinine, aspartate aminotransferase, glutamic pyruvic transaminase, and total bilirubin were significantly lowered after CRRT treatment (p<0.05). Perioperative management of CRRT was safe and effective for severe AMI patients.

## INTRODUCTION

In China, the incidence of acute myocardial infarction (AMI) has been on the rise over the past decade [1]. Timely and effective reperfusion therapy is a powerful treatment strategy, especially for ST-segment elevation myocardial infarction (STEMI) patients. The optimal reperfusion strategy depends on shortening the myocardial ischemia time as soon as possible. Primary percutaneous coronary intervention (PCI) has replaced thrombolysis as the major therapy [2]. Although some patients underwent emergency PCI with target vessel Thrombolysis in Myocardial Infarction (TIMI) flow grade 3, problems such as overlong ischemia time, advanced age, and comorbidity may result in worse cardiac function, systemic deterioration, and even multiple organ failure (MOF) [3]. Furthermore, PCI requires a contrast medium, which may increase the risk of contrast-induced nephropathy (CIN), particularly in patients with chronic kidney disease (CKD). In this context, pharmacotherapy may not be sufficient, and mechanotherapy, such as continuous renal replacement therapy (CRRT), becomes necessary [4].

Severe AMI patients frequently experience multiple comorbidities, including concurrent pulmonary congestion and renal function decline. The heart, lungs, and kidneys are interdependent and can trigger and perpetuate injury in one another through hemodynamic, neurohormonal, and cell signaling feedback mechanisms [5]. In cases of hemodynamic abnormality, CRRT can adjust circulation capacity to ensure adequate blood supply to many organs. Approximately 3%-4% of AMI patients are estimated to require treatment with CRRT or hemodialysis [6].

The perioperative use of CRRT in severe AMI patients is not yet fully understood regarding its safety and efficacy. Therefore, this study aimed to evaluate the prognosis of severe AMI patients who underwent coronary intervention and received perioperative CRRT treatment.

## MATERIAL AND METHODS

We conducted a retrospective, single-center study on consecutive patients with AMI who underwent PCI at the Cardiac Care Unit of the First Hospital of Lanzhou University between January 1, 2016, and December 31, 2017. A total of 945 patients who underwent PCI were screened, and 21 patients who received perioperative CRRT were included in the study. No patients were excluded. The baseline characteristics, procedural data, and in-hospital outcomes were collected and presented in Table 1. The patients were divided into two groups, with one group receiving CRRT and the other not receiving it. The study was conducted in compliance with the principles of the Declaration of Helsinki.

**Table 1.** Demographic and clinical characteristics of the study population

	No CRRT (n=924)	CRRT (n=21)	p value
Male, n	802	15 (1.9%)	0.04
Female, n	122	6 (4.9%)	0.014
Age (yrs)	61±11	67± 10	0.070
Hypertension, n (%)	391 (42.4%)	13 (61.9%)	<0.001
Diabetes mellitus, n (%)	176 (19.2%)	11 (52.4%)	0.014
STEMI, n (%)	775 (83.9%)	13 (62.0%)	0.080
Urgent PCI, n (%)	442 (46.8%)	6 (63.0%)	<0.001
Baseline serum creatinine (μmol/L)	80.2±42.3	198.4±102.6	<0.001
IABP, n (%)	33 (3.6%)	5 (23.8%)	<0.001

### Study design

In this retrospective study, the decision to initiate CRRT was made on a case-by-case basis by the responsible cardiologist based on clinical judgment. The criteria for considering CRRT in critically ill patients during the study period included several factors such as acute kidney injury (AKI) with prolonged oliguria (≥24h), overt heart failure (cardiac function≥Killip II), lack of improvement after medical treatment combined with multiple organ dysfunction, elevated creatinine, severe electrolyte disorder, or metabolic acidosis. The timing of CRRT initiation was categorized as extremely early if started within 24 hours, early if started between 24 to 72 hours, and late if started after 72 hours.

### CRRT methods

For all patients, CRRT was performed using one of three methods: continuous veno-venous hemofiltration (CVVH), continuous veno-venous hemodialysis (CVVHD), or continuous veno-venous hemodiafiltration (CVVHDF), with internal jugular vein access. Different anticoagulant methods were adopted, including intravenous heparin, regional citrate anticoagulation, and citrate combined with heparin. Dialysis and replacement fluid were prepared according to the blood gas analysis results. Additionally, 5% sodium bicarbonate and 5% calcium chloride were infused through the peripheral vein. The blood pump was set to deliver 160–200 ml/min, with a fluid replacement rate of 1800-2500 ml/h, and fluid loss was adjusted according to clinical need. The actual ultrafiltration rate was maintained below 35 ml/kg/h, and the filtration fraction was kept below 25% during CRRT.

### Data collection and assessment

In the CRRT group, all patients were carefully evaluated, and their data were collected prospectively and entered into a computer database. The prognosis was assessed using the TIMI score [7], calculated based on initial clinical history, electrocardiograms, and laboratory values collected upon admission. Renal function was assessed using the modified RIFLE (Risk, Injury, Failure, Loss, End-stage kidney disease) criteria [8]. In patients requiring CRRT, the AKI stage, according to the modified RIFLE criteria, was determined based on the maximum class reached before CRRT initiation. The coronary lesion was assessed by ABC stenosis morphology classification [9].

### Statistical analysis

Categorical variables were expressed as numbers and percentages. Continuous data were presented as mean ± standard deviation (SD) or as median (25 and 75% percentile [IQR]). Differences between groups for continuous variables were analyzed using an unpaired t-test or Kruskal-Wallis H test. One-way repeated measures ANOVA, Friedman repeated measures ANOVA on ranks, or the Bonferroni test was used for longitudinal comparison within the group. Pearson's chi-square or Fisher's exact tests were performed for categorical variables. All statistical tests were 2-sided, and a p-value<0.05 was considered statistically significant. Analyses were performed using IBM SPSS Statistics, version 22, and SAS, version 9.4.

## RESULTS

A total of 945 consecutive patients were included in this retrospective study, of whom 21 required CRRT during their index hospitalization. The baseline clinical characteristics of patients treated with CRRT and those who did not require CRRT are illustrated in Table 1. The CRRT group had a higher proportion of women (p=0.040) and were significantly older (61±11 vs. 67±10, p=0.014) compared to those who did not require CRRT. Patients treated with CRRT were also more likely to have diabetes (19.2% vs. 52.4%, p<0.001) and worse renal function. Moreover, they showed worse cardiac function and needed Intra-Aortic Balloon Pump (IABP) support more frequently (3.6% vs. 23.8%, p<0.001).

The pre-procedural and procedural characteristics of CRRT of the 21 severe AMI patients requiring CRRT are shown in Table 2. All patients were evaluated by TIMI scores and were defined as having intermediate or high cardiovascular risk. The coronary lesions in all patients were B2 or C-type, as classified by ABC stenosis morphology, and 3 of them had lesions in the left main coronary artery. Among the 11 patients with diabetes, 8 had abnormal glycosylated hemoglobin. Of the patients receiving CRRT, 17 (81%) had pulmonary edema, and 8 (38.1%) had combined cardiac shock. Additionally, 9 patients (42.9%) were diagnosed with a ventricular aneurysm, intracardiac thrombus, or ventricular septal rupture through echocardiography. Mechanical ventilation, or IABP, was used in 9 patients (42.9%). Two patients died during hospital treatment.

**Table 2.** Column I. Pre-procedural and procedural characteristics of CRRT-treated patients

Hypertension, n (%)	13 (62.0)
Diabetes mellitus, n (%)	11 (52.4)
Abnormal glycosylated hemoglobin, n (%)	8 (38.1)
Pulmonary edema, n (%)	17 (81.0)
Cardiac shock, n (%)	8 (38.1)
**Echocardiography**	
Ventricular aneurysm, n (%)	5 (23.8)
Intracardiac thrombus, n (%)	2 (9.5)
Ventricular septal rupture, n (%)	2 (9.5)
**Baseline cardiac function**
EF (%)	42.3±6.1
CO (L/min)	5.1±0.8
CI (L/min·m2)	2.9±0.5
**TIMI score**		
High risk, n (%)	14 (66.7)
Intermediate risk, n (%)	7 (33.3)
Low risk, n (%)	0 (0)
**AKI stage prior to CRRT initiation**	
None, n (%)	4 (19.0)
Risk stage, n (%)	8 (38.1)
Injury stage, n (%)	4 (19.0)
Failure stage, n (%)	5 (23.8)
Infarction location	
Anterior + side wall, n (%)	7 (33.3)
Anterior wall, n (%)	4 (19.0)
Inferior wall + right ventricular, n (%)	2 (9.5)
NSTEMI, n (%)	8 (38.1)
**ABC stenosis morphology classification**	
A+B1, n (%)	0 (0)
B2, n (%)	15 (71.4)
C, n (%)	6 (28.6)
Left main disease, n (%)	3 (14.3)
**Time of PCI initiation**
<12h emergency, n (%)	1 (4.8)
>12h emergency, n (%)	8 (38.1)
Selective intervention, n (%)	12 (57.1)
**Time of CRRT initiation**	
Very early, n (%)	6 (28.6)
Early, n (%)	8 (38.1)
Late, n (%)	7 (33.3)

**Table 2.** Column II. Pre-procedural and procedural characteristics of CRRT-treated patients

**CRRT modality**	
CVVH, n (%)	1 (4.8)
CVVHD, n (%)	9 (42.9)
CVVHDF, n (%)	11 (52.4)
**Anticoagulant methods**
Citrate, n (%)	10 (47.6)
Heparin, n (%)	7 (33.3)
Citrate + heparin, n (%)	4 (19.0)
IABP, n (%)	5 (23.8)
Mechanical ventilation, n (%)	4 (19.0)
Bleeding, n (%)	0 (0)
Death, n (%)	2 (9.5)

**AKI** = acute kidney injury; **CRRT = c**ontinuous renal replacement therapy;** PCI** = percutaneous coronary intervention;** CI =** cardiac index; CO = cardiac output;** CVVH** = continuous veno-venous hemofiltration;** CVVHD** = continuous veno-venous hemodialysis; **CVVHDF** = continuous veno-venous hemodiafiltration;** EF** = ejection fraction; **IABP** = intro-aortic balloon pump

The results of the hemodynamic parameters before and after CRRT are shown in Table 3. A significant reduction in the heart rate was found after the treatment (p=0.038). While no significant fluctuation was observed in systolic or diastolic pressure, a reduction trend was observed after CRRT (p>0.05). Most importantly, patients who received CRRT significantly improved their cardiac function (p=0.001, Table 3). Moreover, liver and kidney function indexes were also significantly improved after CRRT (Table 4).

**Table 3.** Comparison of vital signs and cardiac function before and after CRRT treatment

Clinical category	Data	p value
Heart rate (bpm)		
Before CRRT	89±16	0.038
24 h after CRRT	80±15	
48 h after CRRT	84±14	
Systolic pressure (mmHg)		
Before CRRT	120±25	0.535
24 h after CRRT	116±28	
48 h after CRRT	116±19	
Diastolic pressure (mmHg)		
Before CRRT	72±15	0.634
24 h after CRRT	68±10	
48 h after CRRT	69±18	
Cardiac function		
Before CRRT (Killip I/II/III/IV), n	0/5/9/7	0.001
After CRRT (Killip I/II/III/IV), n	2/10/6/3	

**CRRT** = continuous renal replacement therapy

**Table 4.** Comparison of liver and kidney functions before and after CRRT

Category	Before CRRT	24 h After CRRT	48 h After CRRT	P value
Urea nitrogen (mmol/L)	15.5 (10.6, 18.2)	7.5 (6.4, 11.3)	9.9 (7.3, 10.5)	0.003
Creatinine (umol/L)	152.5 (113.3, 233.8)	103.0 (81.3, 192.8)	97.0 (61.5, 162.3)	0.02
Aspartic transaminase (U/L)	240.0 (58.3, 818.8)	69.0 (37.8, 478.8)	71.5 (33.0, 236.0)	0.19
Alanine transaminase (U/L)	172.0 (36.0, 421.3)	128.5 (32.0, 641.5)	123.5 (31.8, 480.3)	0.04
Total bilirubin (μmol/L)	21.8 (17.0, 38.7)	21.7 (12.3, 30.3)	17.6 (12.5, 26.3)	0.009
Direct bilirubin (μmol/L)	6.2 (5.5, 10.3)	6.3 (4.0, 8.1)	5.8 (3.7, 7.1)	0.007
Indirect bilirubin (μmol/L)	15.5 (11.7, 28.5)	16.6 (8.1, 22.5)	11.1 (8.9, 19.9)	0.012

**CRRT** = continuous renal replacement therapy

## DISCUSSION

The prognosis of acute myocardial infarction (AMI) patients has significantly improved due to early diagnosis, advances in therapeutic strategies, and widespread use of emergency operations [10]. However, some patients still experience poor outcomes, often due to delayed opening of blocked vessels, serious coronary lesions, or multiple organ dysfunction. Mechanotherapy has been considered an effective supplement to drug therapy for these severe AMI patients, and some are admitted to the department of nephropathy or the intensive care unit rather than the cardiac care unit. As a result, few studies describe perioperative continuous renal replacement therapy (CRRT) for percutaneous coronary intervention (PCI) in AMI patients. A previous trial with about 2,839 AMI patients found that 83 cases received in-hospital CRRT because of metabolic acidosis, azotemia, hyperkalemia, or oliguria, and the CRRT rate was almost 3.0% [11]. In this study, 2.2% or 21 of 924 AMI patients underwent CRRT, slightly lower than the earlier report.

In this study, female patients accounted for only 13.1%, whereas males accounted for 86.9% of AMI patients. However, a significantly higher proportion of female AMI patients (4.9%) received CRRT than males (1.9%) (p=0.04) (Table 1). The higher rate of CRRT treatment in women may be since AMI in women often presents with a poorer general condition and higher mortality [12]. Furthermore, female patients are more likely to develop multiple organ dysfunction, which may require CRRT as a necessary treatment option. CRRT-treated patients were older in this study (Table 1). The age of acute coronary syndrome cases was positively correlated with mortality, the incidence of cardiogenic shock, and the re-hospitalization rate [13]. Elderly patients with AMI often experience worse liver and kidney function, making CRRT treatment necessary in addition to medication. In addition, the prevalence of diabetes was higher among CRRT-treated patients (Table 1), and most showed abnormal glycosylated hemoglobin.

Poorly controlled frequent hyperglycemia can lead to long-term complications such as kidney failure, and sometimes the creatinine test cannot fully reflect the situation. Long-term diabetes can lead to pain-sensitive nerve damage; therefore, the angina of many diabetic patients is neither typical nor obvious, which can only appear after chest tightness, shortness of breath, or wheezing. Heart attacks can occur without symptoms, resulting in delayed diagnosis and treatment. CRRT treatment provided in the acute phase can improve renal perfusion and promote the metabolism of contrast medium in patients undergoing PCI. More than half (12/21) of the CRRT-treated patients had a normal creatinine level or only AKI stage 1. Abnormal renal function is closely related to myocardial ischemia-reperfusion injury. This is different from other non-cardiovascular disease patients with severe acute illness. In the CRRT group, the utilization rate of IABP was significantly higher, vascular lesions were more complicated, and fewer patients accepted emergency intervention within the time window. These findings are consistent with previous studies [14-16].

The TIMI score is of great significance for identifying high-risk cases in AMI patients [7]. All patients in the CRRT group were defined as having a moderate or high risk by the TIMI risk model. There is a significant correlation between the TIMI risk score for ST-segment elevation myocardial infarction (STEMI) and 30-day mortality, exhibiting a more than 40-fold incremental rise in mortality from individuals with a risk score of 0 to those with a score exceeding 8 [17]. A significant number of patients in the CRRT group showed double-lung texture growing disorder in X-radiograms. It has been confirmed that circulation anomalies can lead to kidney and liver ischemia-reperfusion injury, followed by pulmonary inflammatory response [18,19]. Experimental studies have shown that ischemic acute kidney injury (AKI) is associated with increased pulmonary vascular permeability, cellular apoptosis, and alveolar hemorrhage [20]. Changes in lung texture are often thought to be pneumonia by respiratory physicians, but in reality, it may represent an inflammatory response following multiple organ injuries in severe AMI patients. In this study, the mortality rate was higher in the CRRT group, likely because these patients represented more severe cases with worse prognoses and not necessarily higher mortality associated with CRRT treatment. All patients in the CRRT group were provided with continuous intravenous pumping of heparin and regional citrate anticoagulation in addition to taking oral dual antiplatelet drugs during CRRT. Meanwhile, activated clotting time (ACT) was maintained between 80 and 120 s, or activated partial thromboplastin time (APTT) was kept between 60 and 80 s. No fatal hemorrhage occurred during that period. Hence, combining anticoagulation and antiplatelet therapy is safe, especially under close monitoring of coagulation indexes. Four cases were supplied with both heparin and citrate in our study. The requirement of ACT and APTT was low (ACT:160-180s; APTT:50-60s), and pipeline anticoagulation was provided simultaneously. This approach could reduce the risk of bleeding based on effective anticoagulants. To the best of our knowledge, this finding is novel. However, the sample size in our study was small, and further research is needed to confirm this interesting conclusion.

Severe AMI patients often experience low perfusion due to pump failure or systemic circulation congestion, and cardiogenic shock can sometimes occur. In this study, 38.1% of the CRRT-treated patients had cardiogenic shock. However, after CRRT treatment, the heart rate decreased slightly, and blood pressure was not affected. CRRT has been shown to improve pulmonary congestion more effectively than dialysis [21], and we found that laboratory indexes were well-monitored with CRRT, making it an effective adjunctive treatment for severe MI patients in addition to medication.

This study has several limitations that should be taken into account. First, it is a retrospective study, and the causality between the baseline data and observed outcomes cannot be directly inferred. Second, the sample size of this study was relatively small, and further accumulation of cases is needed to improve the statistical reliability of the results. Third, the study did not analyze multiple factors due to the limited sample size. Lastly, all the CRRT-treated patients in this study had severe conditions and were likelier to have poor cardiac function, chronic renal insufficiency, and even ischemic cardiomyopathy. Therefore, further follow-up is needed to evaluate the long-term prognosis.

## CONCLUSION

Based on the findings of this study, it can be concluded that CRRT may be an effective adjunctive treatment for severe AMI patients, particularly in cases of multiple organ dysfunction and poor renal function. Despite the higher mortality rate in the CRRT group, it is likely that these patients represented more severe cases and had a worse prognosis, and this does not necessarily mean higher mortality associated with CRRT. Anticoagulation with both heparin and citrate may reduce the risk of bleeding, and further research is needed to confirm this approach. However, limitations of the study include small sample size, retrospective nature, and the need for further analysis of multiple factors.
